# Health-Related Quality of Life in Women Carrying Genetic Variants Associated with Breast Cancer Risk: A Descriptive Study

**DOI:** 10.3390/healthcare13222916

**Published:** 2025-11-14

**Authors:** Alejandro Oliva-Muñoz, Manuel Fernández-Alcántara, Nicolás Ruiz-Robledillo, Borja García-Sousa, Hortensia Ballester-Galiana, Silvia Delgado-García

**Affiliations:** 1 Department of Obstetrics and Gynecology, Doctor Balmis General University Hospital of Alicante, 03010 Alicante, Spain; oliva.alej@gmail.com (A.O.-M.); borgarso@gmail.com (B.G.-S.); hortenballester@gmail.com (H.B.-G.); delgadogarciasilvia@gmail.com (S.D.-G.); 2 Faculty of Health Sciences, Department of Health Psychology, University of Alicante, 03690 Alicante, Spain; nicolas.ruiz@ua.es; 3 Breast Cancer Group, Department of Gynecology, Institute for Health and Biomedical Research (ISABIAL), Doctor Balmis General University Hospital of Alicante, 03010 Alicante, Spain

**Keywords:** quality of life, BRCA, genetic counselling, prophylactic surgery

## Abstract

**Background/Objectives**: Breast cancer is the most common cancer among Spanish women. Carriers of certain genetic variants are at increased risk, which can significantly impact their quality of life. The main objective of the present research was to describe the health-related quality of life in women with breast cancer-associated genetic risk variants, distinguishing between those who had already developed cancer and those who did not. Additionally, we aimed to identify the variables influencing the decision to undergo risk-reducing surgery. **Methods**: Descriptive using the questionnaires BREAST-Q, SF-12 and DASS-21. **Results**: A total of 63 women participated, with a mean age of 43.38 years. In the 38.1% the genetic variant was identified during the diagnosis of breast cancer, while the rest did not have cancer. We found significantly lower scores for women with breast cancer in the BREAST-Q modules *Satisfaction with breasts* (*p* = 0.035) and *Physical well-being: chest* (*p* = 0.007), as well as in the physical component of SF-12 questionnaire (*p* = 0.005). Anxiety scores with DASS-21 were significantly higher in breast cancer patients (*p* = 0.017). A total of 55.6% of the patients decided to undergo bilateral prophylactic mastectomy, while 60.31% bilateral adnexectomy. These rates were significantly higher in breast cancer patients (*p* = 0.003), older women (*p* = 0.001), those with at least one child (*p* = 0.002) and those who were already menopausal (*p* = 0.0021). Women who underwent bilateral prophylactic mastectomy reported significantly lower scores in the BREAST-Q modules *Satisfaction with breasts* (*p* = 0.033) and *Physical well-being: chest* (*p* = 0.025), compared to the ones who decided to undergo a follow-up. **Conclusions**: Health-related quality of life is significantly lower in women with pathogenic genetic variants who have developed breast cancer. This may contribute to a higher rate of risk-reducing surgeries in this group.

## 1. Introduction

Breast cancer is the most prevalent among Spanish women and the leading cause of death due to cancer. It typically develops between the ages of 45–65 years [1]. Risk factors are multiple, with only 10–15% considered to be purely hereditary [2]. The most extensively studied genes are *BRCA1* and *BRCA2* [3]. Women carrying a pathogenic variant in either of these genes may benefit from a specific follow-up protocol, as well as from risk-reducing surgeries.

Bilateral mastectomy has been shown to reduce the risk of breast cancer by approximately 90%, while bilateral salpingo-ophorectomy reduces the risk of ovarian, fallopian tube and peritoneal cancer by about 80% [4]. The timing of undergoing salpingo-ophorectomy remains controversial, but it may be considered between the ages of 35–40 years, once reproductive desires have been fulfilled. With both surgeries it is possible to reduce the perception of risk and cancer-related anxiety [5]. However, mastectomy may have a significant impact on body image, and salpingo-ophorectomy can induce iatrogenic menopause in some cases. This may lead to an increased risk of cardiovascular disease, bone fractures, vasomotor symptoms, cognitive changes, and a decrease in sexual desire [6,7,8].

Some studies have investigated the impact of risk-reducing surgeries on health-related quality of life (HRQoL). A systematic review published in 2016 [9] concluded that HRQoL, including mental and physical components, was unaffected after undergoing bilateral mastectomy. These findings are in agreement with subsequent research [10,11,12], particularly with a meta-analysis published in 2023 [13]. This may be explained by the significant reduction in cancer-related anxiety over time following surgery, with around 83% of the women being highly satisfied with their decision [5].

There are two aspects that remain controversial in the literature. One is body image after surgery. Some studies [14,15,16,17,18] report that it is not negatively affected, while others [10,19,20] conclude that there is a significant decrease in body image, with less than 50% of the women being satisfied with the appearance of their breasts. Although body image tends to improve over time, more than 70% of women do not reach the baseline levels prior to surgery.

The other controversial aspect is sexual well-being. While some studies report that it is not affected and the quality of sexual relations reaches baseline within one year after surgery [10,14,16,21,22], others [15,20,23] describe a significant decline, attributed to the loss of nipple sensitivity and the potential local adverse effects of surgery, such as pain, edema and skin complications.

Most of the studies are based on the analysis of HRQoL once women have already undergone surgery, without considering the baseline scores. However, we believe that baseline health status is equally important, which is the reason why we developed this study. It gives a different point of view in comparison to earlier studies, as analyzing baseline scores can allow us to have a more accurate assessment of changes that occur throughout the therapeutic procedure and to identify specific patient profiles, in order to offer more personalized treatment and support shared decision making, regarding the type of surgery and optimal timing for each patient.

The main objective of this study was to describe the HRQoL levels at the time of diagnosis of a genetic variant associated with high risk of breast cancer, comparing healthy women with those who have already developed cancer. We will also assess the role of sociodemographic variables such as age, motherhood and menopausal status in the decision to undergo risk-reducing surgery. It was hypothesized that the HRQoL levels of patients who have already developed breast cancer may be lower, characterized by increased levels of stress and anxiety.

## 2. Materials and Methods

### 2.1. Design

Descriptive observational study was conducted between January 2023 and October 2024 at the Breast Pathology Unit of Doctor Balmis General University Hospital of Alicante.

### 2.2. Participants

A convenience sampling method was used. The inclusion criteria were as follows: women carrying any genetic variant associated with an increased breast cancer risk, who were diagnosed and/or treated at the Doctor Balmis General University Hospital of Alicante, regardless of whether they were healthy or had already developed breast cancer. The only exclusion criteria were being under 18 years old and not filling the questionnaires fully and accurately.

### 2.3. Tools

The sociodemographic and clinical variables evaluated included age, menopausal status, parity, body mass index (BMI), use of hormonal contraceptives, tobacco use, personal history of cardiovascular or breast disease, current health status (whether healthy or already developed breast cancer), family history of breast cancer, type of genetic variant, type of risk-reducing surgery, use of hormone replacement therapy (HRT), and postoperative adverse effects.

For the group of patients who had already developed cancer, we evaluated pathogenic variables such as the affected breast, tumor size, multifocality and multicentricity, TNM staging, histologic type, phenotype, treatment regimen, use of sentinel lymph-node biopsy and its result.

HRQoL was evaluated with three questionnaires.

*BREAST-Q.* It is a tool developed in 2009 [24], based on *Patient Reported Outcome Measures (PROMS)*, which are defined as measures of any aspect related to patient’s health status, directly reported by the patient. The questionnaire is specifically designed to evaluate HRQoL, satisfaction and patient experience following breast surgery, with different modules depending on the type of surgery: augmentation, reduction/mastopexy, breast-conserving surgery, mastectomy and reconstruction. Each module has both preoperative and postoperative versions. In every module there are four thematic scales that can be evaluated independently: psychosocial well-being (10 items), sexual well-being (6 items), satisfaction with breasts (4 items) and physical well-being: chest (10 items). Each scale is scored from 0 (worst) to 100 (best). For this study we used the preoperative version of BREAST-Q 2.0 2017 © in Spanish, under the license of *Memorial Sloan Kettering Cancer Center and the University of British Columbia.* This version was freely downloaded from the QPortfolio website (https://qportfolio.org/breast-q/breast-cancer/ (accessed on 7 December 2022)). It has a Cronbach’s alpha coefficient of 0.877 for the psychosocial well-being scale; 0.902 for sexual well-being; 0.821 for satisfaction with breasts and 0.876 for physical well-being: chest [25]. In 2017, Mundy et al. [26] published the normative scores for each of the preoperative scales (mean ± standard deviation): psychosocial well-being 71 ± 18; sexual well-being: 56 ± 18; satisfaction with breasts: 58 ± 18 and physical well-being: chest 93 ± 11.

*Depression Anxiety and Stress Scale—21 (DASS-21)* [27]. It is a validated tool that explores mental health symptoms. The scale consists of 21 items, with three subscales: depression, anxiety and stress. Each item scores from 0 to 3. The subscales can be evaluated independently by summing the scores of their respective items. A global indicator of emotional symptoms can also be obtained by summing the scores of all items. Higher scores reflect more severe symptomatology. In this study we used the Spanish version, which has a Cronbach’s alpha coefficient of 0.93 for the global score; 0.88 for the depression subscale and 0.83 for anxiety and stress [28]. The reference scores used are as follows: depression: 5–6 mild, 7–10 moderate, 11–13 severe, 14 or more extremely severe; anxiety: 4 mild, 5–7 moderate, 8–9 severe and 10 or more extremely severe; stress: 8–9 mild, 10–12 moderate, 13–16 severe and 17 or more extremely severe [27].

*SF-12 Health Survey* [29]. This is one of the most widely used generic tools for evaluating HRQoL worldwide. It consists of a shortened version of SF-36 survey, comprising 12 items covering eight dimensions: Physical Functioning, Social Functioning, Role-Physical, Role-Emotional, Mental Health, Vitality, Bodily Pain and General Health. Based on these items, two summary scores are calculated: the Physical Component Summatory (PCS) and the Mental Component Summatory (MCS), each representing an overall measure of physical and mental health, respectively. We used an adaptation in Spanish of the original survey [30], which has a Cronbach’s alpha coefficient of 0.85 for PCS and 0.78 for MCS. The reference scores for general population of women between 35 and 55 years of age are between 52.08 and 49.51 for PCS and between 49.50 and 48.86 for MCS [31].

### 2.4. Procedure

Those women who were identified as carriers of genetic variants associated with an increased risk of breast cancer from January 2023 onwards were referred to the Breast Pathology Unit’s outpatient consultations. In the first visit they were widely informed about the possibility of undergoing risk-reducing surgery or following a specific surveillance plan. At the second visit, they communicated their decision regarding surgery. In that moment we informed them about this study and asked for their consent to participate. Those who agreed were asked to complete the HRQoL questionnaires, either on paper in a separate room or online via a Google Forms link that we sent them by email.

This study was approved by the Ethics Committee for Drug Research of Alicante Health Department—General Hospital (CEIm: PI2023-037).

### 2.5. Data Analysis

Qualitative variables were described using frequencies and percentages and comparisons were made using the Chi-square test. For quantitative variables we verified that everyone followed a normal distribution with the Kolmogorov–Smirnov test. They were described using the mean and standard deviation. Groups comparisons were performed using the non-parametric Mann–Whitney U test including 95% confidence intervals. The value of r was used as effect size. Statistical analyses were conducted using SPSS software version 25 RStudio (version 2023) and G*Power version 3.1.

## 3. Results

### 3.1. Description of the Sample

**Figure 1:**
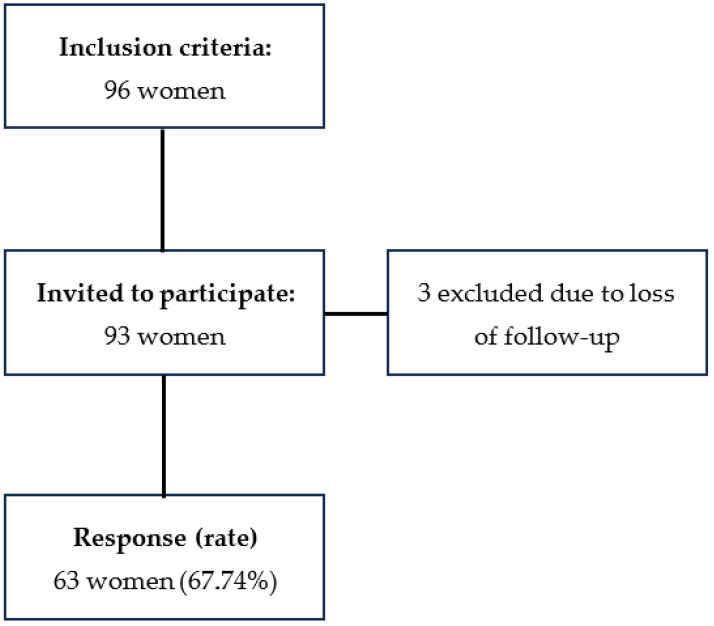
Participant selection process.

A total of 96 women met the inclusion criteria. Three of them were excluded due to the loss of follow-up after diagnosis. Finally, of the remaining 93 women invited to participate in the study, 63 agreed to take part and completed the questionnaires, yielding a response rate of 67.74% (Figure 1).

Table 1 and Table 2 show the main sociodemographic and clinical characteristics of the participants. The sample consisted of young women, most of them premenopausal and had at least one child. In one-third of the cases, the genetic variant was identified during the investigation of an already developed breast cancer, while the rest were asymptomatic regarding breast health. There was a strong family history of breast disease, as most of them reported first-degree relatives affected by breast cancer. The most frequently detected genetic variants were in BRCA2 and BRCA1. Approximately half of the women opted for bilateral mastectomy, whereas the others chose a specific follow-up plan. The majority of patients who underwent surgery received immediate reconstruction, with breast expander placement being the most commonly used technique. Preservation of the nipple–areola complex was possible in more than half of the cases. Nearly two-thirds of the women decided to undergo bilateral adnexectomy, which resulted in iatrogenic menopause in more than half of them. During the course of this study, one patient died due to Creutzfeldt–Jakob disease.

Table 3 presents the pathogenic variables and the treatment regimen received by the subgroup of patients with breast cancer.

The left breast was the most commonly affected, with predominantly localized tumors, most of which were diagnosed at early stages. The most frequent histological type was ductal carcinoma, with a luminal B immunophenotype. More than half of the patients received neoadjuvant chemotherapy, followed by surgery, which was usually bilateral mastectomy with sentinel lymph-node biopsy, being negative most of the times. Half of the patients required radiotherapy, and most completed the treatment with adjuvant chemotherapy, hormone therapy or a combination of both.

### 3.2. Health-Related Quality of Life

Table 4 shows the mean scores obtained from the HRQoL questionnaires for the entire sample and divided into two groups: women with breast and those without. Comparison between groups is presented in the last two columns.

In the BREAST-Q questionnaire, statistically significant differences between the groups were observed in the Satisfaction with breasts (*p* = 0.035) and physical well-being: chest (*p* = 0.007) scales, while no differences were found in the psychosocial and sexual well-being scales.

On the anxiety scale of DASS-21, significantly higher scores were observed in the group of women with breast cancer (*p* = 0.017). Levels of depression and stress were similar between the two groups.

Regarding the SF-12, scores on the PCS were significantly lower in women with breast cancer (*p* = 0.005), as were scores in the Physical Functioning (*p* = 0.006), Role-Physical (*p* = 0.019) and General Health (*p* = 0.004) domains. No differences were found in the MCS or any of its associated domains.

Table 5 presents some of the analyzed variables, comparing women who chose to undergo prophylactic bilateral mastectomy with those who opted for clinical follow-up. The proportion of patients with breast cancer who decided mastectomy was significantly higher (*p* = 0.003).

In the HRQoL tools, we found that those women who decided mastectomy reported significantly lower scores on the Satisfaction with breasts (*p* = 0.033) and physical well-being: chest (*p* = 0.025) scales of the BREAST-Q. No differences were found in the psychosocial and sexual well-being scales. In the DASS-21, we observed higher levels of depression, anxiety and stress in women who chose mastectomy, although the differences were not statistically significant. There were no differences in any of the summaries of the SF-12 or any of its domains, except for Physical Functioning, where scores were lower in the mastectomy group (*p* = 0.019).

Table 6 shows the other analyzed variables, in this case comparing patients that decided to undergo any type of prophylactic surgery (bilateral mastectomy, adnexectomy or both) with those who chose a clinical follow-up. Statistically significant differences were observed in the mean age, with women who chose surgery being older (*p* = 0.001). In line with this, nearly all postmenopausal women opted for a prophylactic surgery (*p* = 0.021). Furthermore, most women who had already had at least one child had undergone one of the surgeries, whereas only half of the nulliparous made this decision, with this difference also reaching statistical significance (*p* = 0.002).

## 4. Discussion

The main objective of this study was to describe the HRQoL scores in women carrying genetic variants associated with an increased risk of breast cancer, comparing those who had already developed cancer with those who had not. The need for this study arises from the growing importance of quality of life and treatment satisfaction nowadays. A thorough understanding of the experiences of affected women may facilitate the provision of more comprehensive preoperative information, thereby enabling patients to make fully informed decisions.

### 4.1. Health-Related Quality of Life

The starting point must be investigating the baseline HRQoL scores, before the treatment. A study published by Mundy et al. [26] established the normative scores of the preoperative scales of BREAST-Q, based on women from the general population. However, subsequent studies have argued that these scores should also be established specifically in women with a recent breast cancer diagnosis, as they consider that they are not comparable to women from the general population due to the significant impact of this disease on quality of life [32,33]. In both studies, the mean scores reported by women with breast cancer do not differ from those of the general population in the psychosocial well-being, sexual well-being and satisfaction with breasts scales. Thus, the authors conclude that a cancer diagnosis does not significantly impact these life domains. However, they report a lower mean score in the physical well-being: chest scale, with a difference of nearly 10 points. To determine whether this difference is significant or not, Voineskos et al. [34] established MDI scores (Minimal Important Difference) for the BREAST-Q questionnaire in 2019. These are defined as the minimal difference in scale scores that can be clinically relevant, perceptible to patients and potentially lead to changes in healthcare. Based on the 0 to 100 BREAST-Q scoring system, the authors fixed an MDI of 4 points for the psychosocial well-being, sexual well-being and satisfaction with breasts scales and an MDI of 3 points for the physical well-being: chest scale. Our results are consistent with these findings from previous literature. We did not observe significant differences between women with and without a cancer diagnosis in the psychosocial and sexual well-being scales, and our mean scores do not differ from the normative values [26].

The scale in which we have found the most significant impairment of quality of life is the physical well-being of the chest. This scale evaluates physical problems related to the breast such as pain, muscle tension and stiffness. Although breast cancer is usually asymptomatic, since it is often diagnosed at early stages, diagnostic tests as mammograms and biopsies can commonly lead to pain, local edema and hematoma. In this regard, Klifto et al. [35] published a study in 2020 in which they compared preoperative quality of life between women with breast cancer and a control group of women without any breast surgery or pathology. Regarding their findings, they observed not only lower scores among women with breast cancer in physical well-being: chest scale but also in the satisfaction with breasts. The reason they propose is that breast cancer is a pathology with a very specific location on a visible organ that carries deep personal and relational significance. Consequently, being aware of having a tumor in such area induces a sense of vulnerability and dissatisfaction with this part of the body. Interestingly, a similar pattern can be observed in our data among women without cancer, who also show a negative impact on this domain compared to the general population. This is because, although they do not have cancer, they carry genetic variants associated with it. As a result, they also undergo diagnostic tests, which can lead to the appearance of symptoms. Moreover, these results suggest that the physical discomfort in this group could not only be a consequence of medical procedures but also of the psychological burden related to the perception of vulnerability and the anticipation of disease, which could amplify bodily sensations, health anxiety and somatic awareness.

In the present study we also employed a generic quality of life assessment tool, the SF-12. The results are consistent with those obtained from the BREAST-Q and with previous studies [36]. Although no significant differences were observed between women with and without breast cancer in the mental health domains, breast cancer had a negative impact on physical health domains, such as Physical Functioning, Role-Physical and General Health, with scores below the normative values of the general population [31].

Although no significant differences were found in the mental health domains of the HRQoL assessment tools, it is possible that these instruments did not capture the specific emotional difficulties experienced by these groups of patients. For this reason, a more specific measure of psychological distress was included in this study. It has been demonstrated that a cancer diagnosis alone can raise anxiety and stress levels. To assess these aspects, we used the DASS-21 scale. We observed that women with breast cancer exhibited significantly higher levels of anxiety, with mean scores corresponding to a moderate degree of severity. According to the literature, this increase in anxiety levels tends to occur particularly in the period between the cancer diagnosis and the initiation of the treatment and may contribute to a heightened perception of physical symptoms in the chest area [37], which is consistent with the findings previously presented in this study.

Gandhi et al. [21] published in 2022 another study in which they observed that those women with clinically relevant levels of anxiety prior to surgery reported poorer scores in terms of satisfaction with the postoperative results, particularly in the psychosocial well-being scale. These findings suggest that pre-existing psychosocial disfunctions are unlikely to be resolved by surgery alone. Therefore, it is essential to identify this patient profile during the preoperative period, as they may benefit from more comprehensive psychosocial intervention.

In this regard, we should reflect deeply on these results and revise our working approach, as they have a great clinical significance. During the initial consultation, when communicating the cancer diagnosis, it is essential to include questions regarding quality of life, particularly physical well-being. If a patient appears to be significantly affected, we should recommend following a specific rehabilitation plan under the supervision of an experienced physiotherapist for breast cancer. Patients should also be informed that the physical symptoms may worsen in the following days, as they process the diagnosis psychologically and undergo additional studies on the breast that may be needed. If we indicate surgery, the timing is crucial. Women with significant impairment of their physical well-being may benefit from postponing the surgery until the moment they feel recovered, as long as the oncological disease allows it. If not, the adverse effects of surgery may have a greater impact.

### 4.2. Decision of Risk-Reducing Surgery

The second objective of this study was to identify which variables may influence the decision to undergo risk-reducing surgery. The most relevant factor was having a recent diagnosis of breast cancer. In the literature it is reported that the women who choose to undergo this type of surgery tend to have higher levels of worry, particularly those who have already developed cancer, as they fear the possibility of developing it in the contralateral breast.

The proportion of prophylactic surgeries among women carrying genetic risk variants has been reported to range between 6 and 47%, with considerable variability across countries [38]. In our study, the proportion of surgeries in the group of women without cancer falls within this range, but we have observed a significantly higher rate in the cancer group.

According to age, we identified a specific patient profile more likely to choose surgery: women of older age, already menopausal, and who have at least one child, suggesting that they have completed their childbearing. This contrasts with the results from other studies [38,39], which report that the highest surgery rates are among younger women under the age of 35. They argue that the benefits of prophylactic mastectomy decrease with age. Furthermore, younger women are generally open-minded to breast reconstruction, whereas older women may be less willing to accept drastic changes in their body image. We believe that our results can be explained by the fact that many women of our cohort decide to undergo both risk-reducing surgeries (mastectomy and adnexectomy) simultaneously. As a result, they wait until they have completed childbearing and are approaching menopause, aiming to avoid severe symptoms associated with the sudden drop in estrogen levels. Another important variable described in the literature is family history. Experiencing the death of a relative due to breast or ovarian cancer often influences the timing of the decision to undergo surgery. In such cases, the decision is frequently linked to the age at which the relative was diagnosed with cancer, rather than the patient’s own age [40]. The fact of having a family is also a relevant factor, particularly having children, as women often seek to avoid, at all costs, the possibility that their children might experience the loss of their mother [39]. Future qualitative studies in this population may provide valuable insights by further exploring about the importance of family bonds in the decision-making process.

We also observed that patients who opted for surgery reported lower satisfaction and reduced physical well-being in the breast area when completing the BREAST-Q. We hypothesize that some of them may perceive surgery as an opportunity to improve their body image. This interpretation suggests that women who choose surgical treatment might already experience greater dissatisfaction or discomfort with their breasts, either due to visible changes associated with the tumor, asymmetry or altered self-perception following diagnosis. Indeed, it has been published that women with lower preoperative self-perception tend to experience greater satisfaction with cosmetic outcomes following breast reconstruction [41].

Finally, regarding anxiety and stress levels, some studies conclude that they are higher in patients who choose surgery [42], while others argue that there are no significant differences at the time of decision making [38], which is consistent with our findings. However, one point on which there is clear consensus in the literature is that anxiety levels decrease following surgery.

Incorporating these results into our daily clinical practice, we consider it important to perform a preoperative psychosocial screening, asking the patients about their expectations regarding surgery. It may be useful to use images of previous patients or 3D virtual simulations, so the women can adapt their expectations to a realistic outcome. After surgery, we should monitor HRQoL levels, repeating similar evaluation protocols after 2, 6, 12 and 24 months, so we can determine if the expectations have been fulfilled and identify those patients that need deeper explanations or a psychosocial intervention by a specialist.

We identified some limitations during the development of this study. These are primarily related to the small sample size and unicentric design, which resulted in some groups being underrepresented in comparative analyses. Also, we did not use repeated testing corrections to reduce Type I error. Moreover, since we worked with voluntary response questionnaires, the presence of response bias should be considered, particularly in sensitive areas such as sexuality and mental health. Recall bias may have also influenced the results, as some questionnaires inquire about past experiences.

## 5. Conclusions

Among women carrying genetic variants associated with an increased risk of breast cancer, those who have already developed the disease report lower HRQoL. This is explained by the impact of the disease on quality of life, particularly on physical well-being because of the side effects of treatments and diagnostic tests and the cancer-related anxiety, which may contribute to a heightened perception of physical symptoms. Moreover, this group of patients undergoes risk-reducing surgeries at a higher rate. This has clinical implications, as we should investigate preoperative HRQoL in order to identify those patients and offer them a personalized treatment.

This study could provide a basis for future investigations adopting a prospective approach. By establishing preoperative baseline HRQoL levels, it would be possible to analyze how quality of life is affected after surgery and to determine whether the patient is satisfied or not with her decision.

Additionally, it would be interesting to further explore the subjective experiences and emotional reactions of women living with this genetic risk. This could be achieved through a qualitative study using individual interviews.

Our findings underscore the importance of early HRQoL assessment in women with pathogenic variants, enabling multidisciplinary teams to offer targeted psychological and physical support before and after preventive surgery.

## Figures and Tables

**Table 1:** Sociodemographic characteristics of the sample.

Variable	Women (n = 63) Mean (SD) or N (%)
Age (years)	43.38 (12.34)
Menopause	
Yes	23 (36.5)
No	40 (63.5)
Age of menopause (years)	47.26 (4.5)
Number of children	
0	20 (37.1)
1	13 (20.6)
2	23 (36.5)
3	5 (7.9)
4	2 (3.2)
Age of first pregnancy (years)	28.28 (6.54)
Time of breastfeeding (months)	10.52 (11.74)
BMI (kg/m^2^)	25.02 (5.59)
Group of BMI	
Normal weight	35 (55.6)
Overweight	16 (25.4)
Obesity class 1	7 (11.1)
Obesity class 2	3 (4.8)
Use of hormonal contraceptives	
Yes	39 (61.9)
No	22 (34.9)
Time of use of hormonal contraceptives (years)	4.5 (5.5)
Tobacco use	
Yes	23 (36.5)
No	40 (63.5)

**Table 2:** Clinical characteristics of the sample.

Variable	Women (n = 63) Mean (SD) or N (%)
Cardiovascular disease	
Yes	18 (28.6)
No	45 (71.4)
Previous breast pathology	
No	39 (61.9)
Benign	14 (22.2)
Of risk	2 (3.2)
Malignant	8 (12.7)
Breast health status	
Healthy	39 (61.9)
Breast cancer	24 (38.1)
First-degree relatives with breast cancer	
Yes	36 (57.1)
No	27 (42.9)
Genetic variant	
BRCA 1	17 (27)
BRCA 2	29 (46)
PALB2	7 (11.1)
ATM	6 (9.5)
Others	4 (6.3)
Risk-reducing surgery	
No	16 (25.4)
Bilateral mastectomy	9 (14.3)
Bilateral adnexectomy	12 (19)
Both	26 (41.3)
Nipple–areola complex preservation	
Yes	20 (54.1)
No	17 (45.9)
Immediate reconstruction	
No	5 (13.5)
Expander	22 (59.5)
Prosthesis	7 (18.9)
Autologous	3 (8.1)
Iatrogenic menopause	
Yes	21 (53.8)
No	18 (46.2)
Hormone replacement therapy	
Yes	5 (13.2)
No	33 (86.8)
Complications	
Seroma	8 (12.7)
Necrosis of tissues	3 (4.8)
Bleeding	1 (1.6)
Pain	3 (4.8)
Death	1 (1.6)

**Table 3:** Pathogenic and treatment variables in the subgroup of breast cancer patients.

Variable		Women with Breast Cancer (n = 24) Mean (SD) or N (%)
Affected breast	Right	9 (37.5)
	Left	11 (45.8)
	Bilateral	4 (16.7)
Multifocal tumor		
Yes		3 (13)
No		20 (87)
Multicentric tumor		
Yes		1 (4.3)
No		22 (95.7)
Lymph node stage (N)		
N0		15 (62.5)
N1		5 (20.8)
N2		2 (8.3)
N3		2 (8.3)
Stage (TNM)		
0 (in situ)		1 (4.2)
IA		7 (29.2)
IB		0 (0)
IIA		9 (37.5)
IIB		3 (15.5)
IIIA		2 (8.3)
IIIB		0 (0)
IIIC		2 (8.3)
IV		0 (0)
Tumor size (mm)		29.25 (22.68)
Histological type		
Ductal		23 (95.8)
Lobular		1 (4.2)
Inmunophenotype		
Luminal A		1 (4.3)
Luminal B		13 (56.5)
Luminal B HER 2 +		1 (4.3)
Pure HER 2		0 (0)
Triple negative		8 (34.8)
Neoadjuvant chemotherapy		
Yes		14 (58.4)
No		10 (41.6)
Type of surgery		
Breast-conserving		8 (33.3)
Unilateral mastectomy		1 (4.2)
Bilateral mastectomy		15 (62.5)
Sentinel lymph-node biopsy		
Yes		20 (83.3)
No		4 (16.7)
Result of sentinel lymph-node biopsy		
Negative		18 (90)
Micrometastasis		0 (0)
Macrometastasis		2 (10)
Axillary lymphadenectomy		
Yes		7 (29.2)
No		17 (70.8)
Radiotherapy		
Yes		11 (50)
No		11 (50)
Adjuvant treatment		
No		8 (34.8)
Chemotherapy (CT)		4 (17.4)
Hormone therapy (HT)		6 (26)
CT + HT		5 (21.6)

**Table 4:** Scores in the HRQoL tools and comparison between breast cancer and non-cancer women.

Tool	Entire Sample (n = 63) Scores: Mean (SD)	Non-Cancer (n = 39) Scores: Mean (SD)	Breast Cancer (n = 24) Scores: Mean (SD)	U Mann– Whitney (95% CI)	*p*	*r* (95% CI)	Sensitivity (Post hoc)
Breast-Q							
Psychosocial	70.14 (24.25)	74.74 (21.21)	62.67 (27.34)	341	0.07	−0.026	0.573
Well-being				(202.52;479.48)		(−0.449: 0.023)	
Sexual	63.21 (22.7)	66.62 (18.68)	57.67 (27.59)	372	0.173	−0.168	0.408
Well-being				(235.52;512.48)		(−0.349; 0.084)	
Satisfaction with Breasts	68.14 (24.18)	73.33 (22.53)	59.71 (24.86)	321 (182.52;459.48)	0.035	−0.262 (−0.479; 0.15)	0.688
Physical Well-being: Chest	79.38 (23.18)	85.67 (19.91)	69.17 (24.81)	284.5 (146.02;422.98)	0.007	−0.327 (0.532; 0.086)	0.861
DASS−21							
Depression	4.25 (5.15)	3.44 (4.9)	5.58 (5.36)	344 (205.52;482.48)	0.074	−0.221 (0.445; 0.028)	0.462
Anxiety	3.87 (4.71)	3 (4.42)	5.29 (4.91)	303 (164.52;441.48)	0.017	−0.294 (−0.505; 0.05)	0.571
Stress	5.73 (5.12)	5.23 (5.02)	6.54 (5.18)	383 (244.52;521.48)	0.226	−0.152 (−0.385; 0.1)	0.245
SF−12							
Physical Component Summatory	48 (10.3)	51.02 (8.55)	43.16 (11.21)	271 (132.52;409.48)	0.005	−0.351 (−0.55; −0.113)	0.901
Mental Component Summatory	42.7 (13.46)	44.08 (13.14)	40.47 (13.97)	392 (253.52;530.48)	0.282	−0.136 (−0.371; 0.116)	0.256
Physical Functioning	78.97 (29.85)	87.18 (22.85)	65.63 (35.21)	295.5 (157.02;433.98)	0.006	−0.308 (−0.516; 0.065)	0.854
Role-Physical	68.25 (45.17)	78.21 (41.03)	52.08 (47.73)	329.5 (157.02;433.98)	0.019	−0.308 (−0.516; 0.065)	0.706
Bodily Pain	81.75 (27.39)	86.54 (22.83)	73.96 (32.54)	366.5 (228.02;504.98)	0.099	−0.181 (−0.41; 0.07)	0.508
General Health	53.57 (20.51)	59.62 (18.69)	43.75 (19.85)	280.5 (42.02;418.98)	0.004	−0.334 (−0.538; 0.094)	0.922
Vitality	56.19 (27.32)	59.49 (26.15)	50.83 (28.88)	384.5 (246.02;522.98)	0.225	−0.149 (−0.383; 0.103)	0.317
Social Functioning	72.62 (29)	78.21 (25.76)	63.54 (32.12)	348 (209.52;486.48)	0.074	−0.214 (−0.439; 0.036)	0.591
Role-Emotional	69.05 (45.29)	76.92 (41.11)	56.25 (49.59)	367.5 (229.02;505.98	0.085	−0.179 (−0.409; 0.072)	0.518
Mental Health	64.76 (19.91)	67.19 (19.46)	60.83 (20.41)	386 (247.52;524.48)	0.24	−0.146 (−0.38; 0.105)	0.323

**Table 5:** Variable analysis and scores obtained in the HRQoL tools comparing between bilateral prophylactic mastectomy and follow-up groups.

Variable/Tool	Mastectomy Yes (n = 35) Mean (SD) or N (%)	Mastectomy No (n = 28) Mean (SD) or N (%)	U Mann–Whitney/Chi^2^ (95% CI)	*p*	*r*/*Odds Ratio* (95% CI)	Sensitivity (Post hoc)
Breast health status			8.753	0.003	0.188 (0.057; 0.592)	0.758
Healthy	16 (41)	23 (59)				
Breast Cancer	19 (79.2)	5 (20.8)				
Breast-Q						
Psychosocial Well-being	66.03 (25.22)	75.29 (22.36)	382.5 (240.8;524.2)	0.134	−0.187 (−0.416; 0.063)	0.434
Sexual Well-being	59.51 (24.67)	67.82 (19.42)	401.5 (259.8;543.2)	0.219	−0.154 (−0.387; 0.097)	0.413
Satisfaction with Breasts	61.91 (23.3)	75.93 (23.37)	338 (196.3;479.7)	0.033	−0.265 (−0.481; −0.018)	0.740
Physical Well-being: Chest	73.51 (25.49)	86/71 (17.77)	333 (191.3;474.7)	0.025	−0.274 (−0.488; −0.028)	0.740
DASS-21						
Depression	5.51 (5.87)	2.68 (3.59)	351 (209.3;492.7)	0.051	−0.242 (−0.462; 0.006)	0.715
Anxiety	4.77 (5.45)	2.75 (3.35)	413 (271.3;554.7)	0.282	−0.134 (−0.37; 0.117)	0.522
Stress	6.83 (5.49)	4.36 (4.34)	356 (214.3;497.7)	0.062	−0.234 (−0.455; 0.015)	0.601
SF-12						
Physical Component Summatory	46.24 (11.15)	50.26 (8.83)	393 (251.3;534.7)	0.18	−0.169 (−0.4; 0.082)	0.451
Mental Component Summatory	40.36 (12.59)	45.64 (14.1)	356 (214.3;497.7)	0.064	−0.234 (−0.455; 0.015)	0.444
Physical Functioning	72.14 (31.95)	87.5 (25)	338 (196.3;479.7)	0.019	−0.265 (−0.481; −0.018)	0.653
Role-Physical	61.43 (47.1)	76.79 (41.9)	405.5 (263.8;547.2)	0.163	−0.147 (−0.381; 0.104)	0.369
Bodily Pain	77.14 (31.14)	87.5 (20.97)	415 (273.3;556.7)	0.233	−0.131 (−0.367; 0.121)	0.437
General Health	50.71 (22.27)	57.14 (17.82)	420 (278.3;561.7)	0.293	−0.122 (−0.359; 0.13)	0.333
Vitality	52 (27.95)	61.43 (26.07)	397.5 (255.8;539.2)	0.189	−0.161 (−0.393; 0.09)	0.376
Social Functioning	67.14 (31.37)	79.46 (24.58)	378.5 (236.8;520.2)	0.105	−0.194 (−0.422; 0.056)	0.508
Role-Emotional	61.43 (48.64)	78.57 (39.51)	406 (264.3;547.7)	0.159	−0.146 (−0.38; 0.105)	0.431
Mental Health	60.29 (19.48)	70.36 (19.34)	363.5 (221.8;505.2)	0.072	−0.22 (−0.444; 0.029)	0.629

**Table 6:** Variable analysis comparing any risk-reducing surgery and follow-up groups.

Variable	Surgery Yes (n = 47) Mean (SD) or N (%)	Surgery No (n = 16) Mean (SD) or N (%)	U Mann–Whitney/Chi^2^ (95% CI)	*p*	*r*/*Odds Ratio* (95% CI)	Sensitivity (Post hoc)
Age (years)	46.21 (11.18)	35.06 (11.7)	167 (42.87;291.13)	0.001	−0.416 (−0.602; −0.187)	0.945
At least one child			9.36	0.002	6.17 (1.803; 21.093)	0.787
Yes	37 (86)	6 (14)				
No	10 (50)	10 (50)				
Menopausal status			5.33	0.021	0.177 (0.036; 0.867)	0.531
Yes	21 (91.3)	2 (8.7)				
No	26 (65)	14 (35)				

## Data Availability

The data presented in this study are available upon request from the corresponding author due to privacy preservation.
